# The association of social media with dietary behaviors among adults in the United Arab Emirates

**DOI:** 10.1016/j.heliyon.2024.e35574

**Published:** 2024-07-31

**Authors:** Leila Cheikh Ismail, Tareq M. Osaili, Farah Naja, Mary Wartanian, Gadeer Elkabat, Mariam Arnous, Hala Alkoukou, Maysm N. Mohamad, Sheima T. Saleh, Rameez Al Daour, Emad Masuadi, Habiba I. Ali, Lily Stojanovska, Ayesha S. Al Dhaheri

**Affiliations:** a Department of Clinical Nutrition and Dietetics, College of Health Sciences, University of Sharjah, Sharjah, 27272, United Arab Emirates; b Nuffield Department of Women's & Reproductive Health, University of Oxford, Oxford, OX1 2JD, UK; c Department of Nutrition and Food Technology, Faculty of Agriculture, Jordan University of Science and Technology, Irbid, 22110, Jordan; d Department of Nutrition and Health, College of Medicine and Health Sciences, United Arab Emirates University, Al Ain, 15551, United Arab Emirates; e Department of Public Health Institute, College of Medicine and Health Sciences, United Arab Emirates University, Al Ain, 15551, United Arab Emirates; f Institute for Health and Sport, Victoria University, Melbourne, VIC, 3011, Australia

**Keywords:** Social media, Eating habits, Lifestyle, Diet, Social networking sites, Social media platforms

## Abstract

**Background:**

Social media is an online community that offers a digital setting where people create, share, and access a wide range of information, knowledge, and viewpoints. This study assessed the association between social media use and eating behaviors and whether sociodemographic characteristics and lifestyle habits are correlated with this association. In addition, it assessed whether this effect is different according to changes in lifestyle habits due to the COVID-19 pandemic among adults in the United Arab Emirates (UAE).

**Methodology:**

A cross-sectional web-based study was conducted among 1056 adults living in the UAE. Information on sociodemographic characteristics, social media use, and dietary habits were collected. The Scale of Effects of Social Media on Eating Behavior (SESMEB) was used and a total score ranging from 18 to 90 was generated with higher scores corresponding to a greater effect. The general linear model analysis assessed associations of certain characteristics with the score. Independent T-test and one-way ANOVA test were used to investigate differences based on changes in lifestyle habits due to COVID-19.

**Results:**

Most participants (80.3 %) reported using social media >2 h/day. The mean score was 44.15 ± 12.68 (range 18–90). Increasing age, being a male, spending less time on social media, and not following influencers were associated with lower SESMEB scores. Not consuming breakfast and spending more time on screens for leisure were associated with higher scores (p < 0.05). Significantly higher scores were recorded for those previously infected with COVID-19 and who reported an increase in screen time, food intake, body weight, and meals/day (p < 0.05).

**Conclusion:**

Social media appears to have an association with adults’ dietary habits in the UAE. Spending more time on social media, being a female, and having more screen time were associated with a higher impact. Targeted programs are needed to increase awareness and advocate for a positive lifestyle with social media use.

## 1 Background

Social media is an online community that offers a virtual setting where people can create and share information, connect and interact with each other, and exchange and access a wealth of information, knowledge, and viewpoints [1]. The advancements in information and communication technology have made the use of the internet and, subsequently, social media platforms (SMP) an integral part of daily lives [2]. Within the Internet environment, people can establish social links and interact with people around the globe via SMP. According to the latest statistics, 64.6 % of the global population are internet users and 59.9 % are social media users [3].

In particular, the Middle East is thought to be a market leader in social media use, with many countries ranking above the global average of social media use. For instance, the United Arab Emirates (UAE) ranks first in social media use, followed by Bahrain, Qatar, Lebanon, and Oman (90.5%–100 % social media users) [4]. Moreover, the time spent on social media has increased over the years, with an average of 2.5 h globally and 3.25 h in countries such as the UAE and Saudi Arabia [4].

The use of SMP, particularly among the youth, has highlighted the cruciality of investigating its impact on health. Available evidence indicates that the use of SMP impacts adolescents' sleep and eating behavior, mediating their effects on overall health [5]. Within SMP, the algorithms moderate and facilitate what users view and what content is promoted considering users' interests and attitudes [6]. This way, SMP provides personalized content to users and enhances their experience [6]. However, it may significantly impact people's social perceptions by restricting what they see and exacerbating the relationship between social media and negative impacts on health and well-being, and exacerbating [7,8].

The constant presence, inclusion, and exposure of food on these platforms can act as a cue and trigger people to eat [9], diminishing individual interoceptive awareness and limiting their ability to respond to physiological hunger and satiety signals [10,11]. This external influence, such as indirect exposure to food, which is usually energy-dense, through social media may result in an increased desire to eat [[12], [13], [14]], and with constant exposure along with low interoceptive awareness, people may overconsume foods and adopt unhealthy eating habits leading to health-related problems, particularly obesity [15].

‘Social media influencers’ represent another important factor impacting people's lives and lifestyle habits in the present time [16,17] by advocating for different products or ideas and indirectly shaping their audience's behavior through posts and videos [18]. It has also been shown that these influencers mainly promote restaurants that serve high-energy-dense foods compared with restaurants that serve better healthy choices [19]. This also includes the food producers who use social media and influence their marketing strategies to market their food products, which are usually energy-dense [20,21]. With that being said, SMP can play a major role in eating disorders and negative body image among users [22]. Users are, on one side, bombarded with external cues possibly encouraging unhealthy food choices and on the other side, on one side bombarded with external cues possibly encouraging unhealthy food choices, and on the other side, pressured to maintain a low weight and have an ideal body image to feel included and accepted. Thus, it may lead to low self-esteem and a higher perception of negative body image [23,24].

During the lockdown implemented during COVID-19, the global population has experienced significant changes in daily living, including social life and work systems, and a surge in the use of social media to communicate and obtain information [25]. This impacted the physical and mental health of individuals, playing an important role in shaping people's dietary habits during the pandemic, which has been observed globally and in the Middle East [26,27]. In particular, research amid the COVID-19 pandemic has emphasized its significant impact on individuals' behaviors, particularly their use of social media platforms. A study conducted by Brailovskaia et al. investigated the relationship between the COVID-19 crisis, addictive social media use, and factors such as a sense of control and anxiety symptoms. The findings revealed a positive association between the burden of the COVID-19 crisis and addictive social media use, suggesting that individuals may resort to social media platforms as a coping mechanism during times of uncertainty and stress [28].

Changes in people's lifestyles extended beyond easing the restrictions and the dwindling of COVID-19 infected cases [29,30]. This, in turn, could have inflated the dependence on SMP, increased their use, and exacerbated possible impacts on people's dietary behavior and overall health.

Investigating the effects of social media on dietary habits is crucial in understanding how online communication and information influence people's eating behaviors. Given the limited research on this topic in the UAE, our study aimed to assess the impact of social media on current eating behaviors among adults in this country. Additionally, we aimed to determine whether specific sociodemographic characteristics and current lifestyle habits are associated with this impact. Moreover, given the established relationship between the COVID-19 pandemic and the use of SMP, a secondary objective was to investigate if differences in the effect of social media on eating behaviors exist according to reported changes in lifestyle and dietary habits due to the COVID-19 pandemic.

## 2 **Methods**

### 2.1 Study design and subjects

A web-based, cross-sectional study was conducted in the UAE between January and May 2023. Inclusion criteria were adults (≥18 years) and social media users who live in the UAE. Using Cochran's formula for observational studies, the minimum sample size required was calculated based on the following equation with a confidence interval of 95 %:

$$N=z^{2}\times P\times \left(1-P\right)/e^{2}$$

Where z = 1.96; P = (estimated proportion of the population that presents the characteristic) = 0.5; e (margin of error) = 0.05; N (sample size) = 384 participants, plus 20 % (attrition rate) = 460 participants. A total of 1056 adults completed the online survey which exceeded the calculated sample size due to a high response rate and resulted in an increased statistical power and enhanced external validity.

Participants were recruited using convenient snowball sampling to ensure large-scale recruitment. Adults in the country were invited to participate in the study through the electronic distribution of the survey link via email invitations through the research team contacts, or posts on different SMP, i.e. LinkedIn™, Facebook™, Instagram™, TikTok™, and WhatsApp™. In addition, adults were physically approached at several locations (i.e. shopping centers and coffee shops) and handed a card that included a quick response code (QR) for the electronic questionnaire. Furthermore, interested participants were encouraged to share the link with their contacts. This approach facilitated quick and large-scale dissemination of the survey link and the inclusion of eligible participants. Participants were advised that they could only submit one response using the provided link. The survey platform enforced this limitation to maintain data integrity, allowing only one response per device. The study obtained ethical approval from the University of Sharjah Research Ethics Committee (REC-23-03-22-01-S) and written informed consent was obtained from all participants.

### 2.2 Study questionnaire and data collection

A self-administered multi-component web-based questionnaire was developed by the research team upon a comprehensive review of available literature [29,31,32]. The survey included five sections; sociodemographic information, trends of social media use, dietary and lifestyle habits, the Scale of Effects of Social Media on Eating Behavior (SESMEB), and changes in lifestyle habits due to COVID-19. The sociodemographic section included questions on gender, age, emirate of residence, nationality, marital status, number of children (if applicable), education level, employment status, disease condition, and self-reported weight (kg) and height (cm).

The trends of social media use section was adapted from previously published studies [25,32] which inquired about the time spent on SMP, the rationale of use, and the most used media platforms. It also inquired about the type of information participants look for about food, whether they are interested in nutrition news on social media, whether they follow influencers on social media, and whether social media influencers might affect their food choices.

The dietary and lifestyle habits section was adapted from recently published studies in the region [27,28], including questions on the current nature of most consumed meals, and number of meals and snacks consumed per day, whether participants consume breakfast on most days, and whether they skip meals and the reasons if they do, and their water intake. A short food frequency table was provided with 9 different items including fruits, vegetables, milk and milk products, meat/chicken/fish, and sweet drinks, to name a few, with response options of (Never-1-4 times/week-Once/day-2-3 times/day-4 or more times/day). Moreover, other questions inquired about the time spent on exercising, household chores, use of computers for either work or study and use of TV/computer/social media for entertainment.

The next section included the Scale of Effects of Social Media on Eating Behavior (SESMEB) [31], which is a validated and reliable scale with a reliability coefficient of 0.928 used to assess the association of social media with eating behavior. The scale consisted of 18 items related to social media and eating behavior with response options rated on a five-point frequency scale ranging from 1; Never to 5; Always. A total score can be calculated based on the participant's response to each item. The score could range from 18 to 90 with a higher score indicating a higher impact on eating behavior.

The last section was added pertaining to our secondary objective and included questions on changes in dietary habits due to COVID-19 [29] and inquired whether the participants had been infected with covid-19 virus and if they were hospitalized as a result. Participants also were asked if their body weight, food intake, the number of meals, physical activity level, and screen time had decreased, increased, or remained unchanged due to the pandemic.

The questionnaire was first prepared in English and then translated to Arabic and back-translated to English by two independent bilingual researchers from the research team to ensure parallel-form reliability. The entire survey was made available to participants in both languages. A pilot test was conducted on 25 participants to ensure questions clarity before the launch of the online survey. The data from the pilot testing was not included in the study analysis.

The questionnaire was prepared using Google Forms and a Uniform Resource Locator (URL) was generated to send to eligible participants. On the first page of the online survey, an information sheet including the research protocol and objectives was available for participants informing them of their right to withdraw at any point without consequences and ensuring them of their voluntary participation and the anonymous nature of the study. Screening questions were added to ensure that participants were within the inclusion criteria of being 18 years and above, currently living in the UAE and active users of social media. The next page contained an informed consent to which participants were required to check the “Agree” boxes to confirm reading the information sheet and their willingness to participate in the study. Consenting participants proceeded with the survey questions. The survey completion duration ranged from 5 to 10 min, and all questions were required to be answered.

### 2.3 Data analysis

Continuous data were expressed as means ± standard deviations (SD) and categorical data were expressed as counts and percentages. Body mass index (BMI) was calculated using the self-reported weight (in kilograms) divided by the height squared (in meters). The WHO guidelines for BMI classification were used to categorize the BMI into; Underweight (<18.5 kg/m2), Normal (18.5–24.9 kg/m2), Overweight (25–29.9 kg/m2), and Obese (≥30 kg/m2) [33]. A total score for the SESMEB was calculated for each participant based on their responses to the 18 items in the SESMEB [31]. During the data analysis, the score was transformed using a minimum-maximum scaling approach to normalize the data and convert it to a percentage (out of 100). A general linear model analysis was performed to investigate whether certain characteristics (independent variables) can predict the association between social media and eating habits (score as dependent variable). Independent T-test and One-way ANOVA test were used to investigate if the SESMEB score differed based on different effects of the COVID-19 pandemic on certain lifestyle and dietary habits. P values at <0.05 were considered statistically significant. Data were analyzed using SPSS software, version 26.0 (SPSS, Chicago, IL, USA).

## 3 Results

### 3.1 Study participants characteristics

A total of 1056 participants took part in the study, and their characteristics are presented in Table 1. Almost similar proportions of males and females were recruited (52.8 % and 47.2 % respectively). About half of the participants were young adults, with 28.9 % in the 18–25 years group, and 21.9 % in the 34–41 years group. The majority of the participants were Arabs (88.4 %). Participants mainly lived in the three major cities of the UAE with 29.5 % in Abu Dhabi, 28.8 % in Sharjah, and 24.9 % in Dubai. Almost half of the participants (55.3 %) were married. The participants were mostly bachelor's degree holders (57.1 %), and 59.7 % of them were employed. Most participants were classified as having normal weight (35.0 %), followed by overweight (33.9 %) and obese (24 %).

**Table 1:** Characteristics of the study participants (n = 1056).

Characteristics	n	%
Sex		
Male	558	52.8
Female	498	47.2
**Age (years)**		
18–25	305	28.9
26–33	215	20.4
34-41	231	21.9
42-49	179	17.0
>50	126	11.9
**Nationality**		
GCC countries^a^	618	29.9
Arab (other countries)	123	58.5
Non-Arab	315	11.6
**Emirate of residence**		
Abu Dhabi	312	29.5
Dubai	263	24.9
Sharjah	304	28.8
Northern Emirates^b^	177	16.8
**Marital status**		
Married	584	55.3
Single	438	41.5
Divorced/Widowed	34	3.2
**Education level**		
High school or less	157	14.9
College/Diploma	188	17.8
Bachelor's degree	603	57.1
Higher education MSc./Ph.D.	108	10.2
**Employment status**		
Employed	631	59.7
Student	180	17.0
Unemployed	245	23.2
**BMI categories (kg/m**^**2**^**)**		
Underweight (<18.5 kg/m2)	75	7.1
Normal (18.5–24.9 kg/m2)	370	35.0
Overweight (25–29.9 kg/m2)	358	33.9
Obese (≥30 kg/m2)	253	24.0

a GCC; Gulf Cooperation Council countries (UAE, Bahrain, Kuwait, Oman, Qatar, Saudi Arabia).

b Northern Emirates (Ajman, Ras Al Khaimah, Umm Al Quwain, Fujairah).

### 3.2 Trends in social media use

**Fig. 1:**
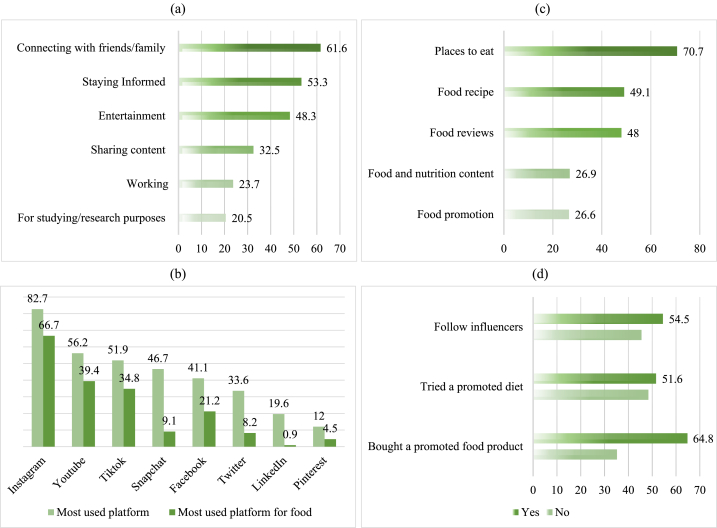
Trends of social media use among study participants. (a) The main use of social media (Multiple responses were allowed); (b) Most used social media platform on a regular basis and in relation to food; (c) Type of information for food searched on social media (Multiple responses were allowed); (d) Following influences and their effect on participant habits.

An overview of social media use among the study participants is shown in Table 2. Most of the participants (80.3 %) reported using social media more than 2 h per day mainly to connect with friends and family (61.6 %), followed by staying informed (53.3 %) and for entertainment purposes (48.3 %) (Fig. 1a).

**Table 2:** Trends of social media use among the study participants (n = 1056).

Characteristics	*n*	%
Time spent on social media use		
≤2 h/day	208	19.7
>2 h/day	848	80.3
**Interest in nutrition on social media**		
Yes	359	34.0
No	298	28.2
Partially	399	37.8
**Reason for following nutrition news on social media (n=758)**^a^		
Learning to eat healthier food	553	52.4
Weight control and weight loss	503	47.6
Learning healthy recipes	439	41.6
To follow current information	340	32.2
Learning to eat in diseases	324	30.7
**Main source of referral in case of a nutrition-related problem**		
Internet/Social Media	445	42.1
Dietitian	359	34.0
Doctor	207	19.6
Food Engineer/specialist	29	2.7
Family/Friends	10	0.9
Other	6	0.6
**Do you pay attention to the sources of nutrition-related posts on social media?**		
Yes	345	32.7
No	309	29.3
Sometimes	402	38.1
**The factor influencing the reliability of nutrition-related posts on social media**		
Written by a nutritionist/dietician	608	57.6
Written by the doctor or healthcare professionals	231	21.9
Written by someone who shares their experiences (losing weight, fighting disease, etc.)	208	19.7
Others	9	0.8

a Multiple responses were allowed.

Among the numerous SMP, Instagram was the most widely used (82.7 %), followed by YouTube (56.2 %) and TikTok (51.9 %) (Fig. 1b). Instagram was also considered the most widely used social media platform to look up food references (66.7 %), followed by YouTube (39.4 %) and TikTok (34.8 %) (Fig. 1b). When asked about what type of food-related information they looked up, most participants used these platforms to find places to eat (70.7 %), search for recipes (49.1 %), and to read food reviews (48 %) (Fig. 1c).

Most participants either had a complete (34.0 %) or partial interest (37.8 %) in nutrition on social media out of which 52.4 %, 47.6 %, and 41.6 % had an interest in learning to eat healthier food or learning to control weight, and weight loss or learning healthy recipes respectively. It was also shown that almost half (42.1 %) of the participants used the Internet and social media as a main source of reference to nutritional problems with a lesser proportion (34.0 %) relying on dietitians. The findings also showed that only 32.7 % of the participants considered the source of any nutrition-related post on SMP. Favorably, the most influential factor in the reliability of nutrition-related posts on social media was the fact that they would be provided by a nutritionist/dietitian (57.3 %).

More than half of the participants (54.5 %) reported following influencers on social media. Two further questions were asked to understand if following influencers can affect certain dietary habits. Around half of the participants reported ever trying a promoted diet by social media influencers (51.6 %) and a higher proportion reported purchasing food products promoted on social media (64.8 %) as shown in Fig. 1c.

### 3.3 Current dietary and lifestyle habits

The dietary habits of the participants are presented in Table 3. Most of the participants consumed homemade meals (70.9 %). Similar proportions of the participants had two or three meals per day (43.6 % and 43.7 % respectively). Furthermore, half of the participants reported having breakfast on most days (50.8 %). More than half of the participants reported skipping meals during the day (64.8 %) for several reasons, mainly due to lack of time (35.1 %). Less than half of the participants reported eating two or one snacks between meals daily (41.1 % and 35.8 % respectively). In terms of water consumption, less than half of the participants consumed the required amount of water daily (45.6 %).

**Table 3:** Eating habits among study participants (n = 1056).

Variables	n	%
Most consumed meals during the week		
Homemade	749	70.9
Fast food	151	14.3
Restaurants	93	8.8
Frozen ready-to-eat meals	40	3.8
Healthy food restaurants	23	2.2
**Number of meals per day**		
1	73	6.9
2	460	43.6
3	461	43.7
4	52	4.9
5 or more	10	0.9
**Eating breakfast on most days**		
Yes	536	50.8
No	520	49.2
**Skipping meals**		
Yes	684	64.8
No	372	35.2
**Reasons for skipping meals (n=684)**		
Lack of time	371	35.1
Lack of appetite	127	12.0
To lose weight	87	8.2
To reduce food intake	66	6.3
Fasting	30	2.8
**Number of snacks between meals per day**		
0	94	8.9
1	378	35.8
2	434	41.1
3	111	10.5
4 or more	39	3.9
**Amount of water consumed per day**		
Less than 8 cups (<2 L)	574	54.4
More than 8 cups (>2 L)	482	45.6

**Fig. 2:**
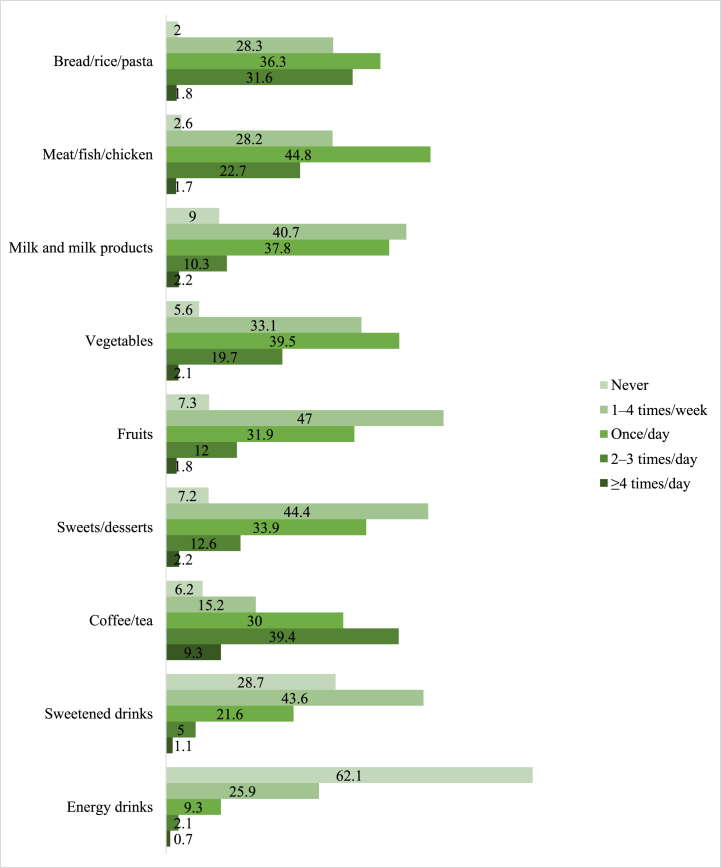
Frequency of consumption of particular foods.

The frequency of consumption of particular foods among the participants is depicted in Fig. 2. In terms of major food groups, most participants reported consuming carbohydrate sources (bread/rice/pasta) and protein sources (meat/fish/chicken) at least once daily (69.7 % and 69.2 % respectively). Most participants consume vegetables daily (61.3 %), while less than half (45.7 %) consume fruits daily. Only half of the participants consume milk and dairy products at least once daily (50.3 %). Caffeinated beverages were popular among participants as the majority reported consuming them at least once daily (78.6 %). Sweetened drinks and energy drinks were less popular as only 27.7 % and 12.0 % consume them daily. Sweets and desserts were consumed by less than half of the participants at least once a day (48.4 %).

**Fig. 3:**
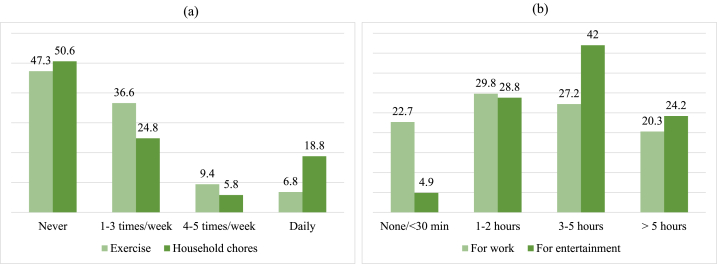
Physical activity and screen time among participants. (a) Physical activity and household chores frequency; (b) Time spent using screen for work and entertainment.

Fig. 3 presents the frequency of physical activity and screen time spent for work or leisure among study participants. Almost half of the participants reported never engaging in any physical activity (47.3 %) and less than 10 % of the participants exercise most days of the week (Fig. 3a). Half of the participants (50.5 %) reported not doing any household chores. Participants mostly spent 1–2 h and 3–5 h daily using screens for work (29.8 % and 27.2 % respectively) (Fig. 3b). For entertainment purposes, most participants reported spending 3–5 h per day on television, computer, and social media (42.0 %) (Fig. 3b).

### 3.4 The scale of effects of social media on eating behavior

**Fig. 4:**
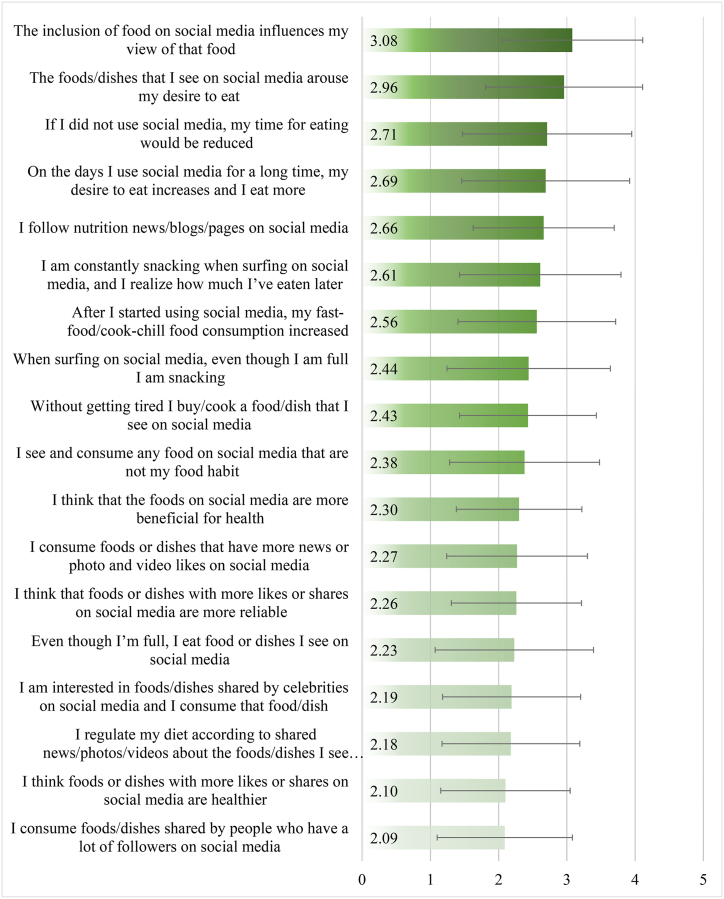
Mean responses to the Scale of Effects of Social Media on Eating Behavior (SESMEB).

Fig. 4 illustrates the mean value for participants' ratings ranging from “1: Never; 2: Rarely; 3: Sometimes; 4: Often; 5: Always” on the 18 items used to measure the effect of social media on their eating behavior. Participants' views about foods were moderately affected by the inclusion of food on social media (3.08 ± 1.03) and their presence on these platforms increased the desire to eat these foods (2.95 ± 1.15). This provides insights into how exposure to food-related content on social media may have a distinct impact on people's perception of and stimulation toward different food items. In addition, participants reported that in case of abstaining from social media, the time allocated for eating will be reduced (2.71 ± 1.24), indicating how social media use may lead to longer meal durations. On the other hand, participants less frequently reported that their food consumption is affected by foods shared by people who have a lot of followers on these platforms (2.08 ± 0.99), the higher likes and shares on food-related posts (2.09 ± 0.94), or those shared by celebrities (2.18 ± 1.00). The mean score of all the items was 44.15 ± 12.68 (range 18–90). Upon data transformation, the mean score corresponded to 36.32 %.

#### 3.4.1 Association between social media's effects on eating behavior and sociodemographic characteristics

Table 4 shows the association between several sociodemographic and social media use factors and the SESMEB score using general linear model analyses. The analysis revealed that the SESMEB score was significantly lower by 0.3 % among older participants (B = −0.3, 95 % CI;-0.4 to −0.1), p < 0.001), by 5.0 % among males (B = −5.0, 95 % CI: 7.6 to −2.5), p < 0.001), by 9.5 % among those who use social media <2 h per day (B = −9.5m 95 % CI: 11.9 to −7.0) and by 2.5 % among those who reported not following influencers (B = −2.5, 95 % CI: 4.7 to −0.4, p = 0.022) compared to their counterparts. Similarly, participants’ scores from GCC countries and other Arab countries were significantly lower than non-Arabs by 5.8 % and 7.4 % respectively (B = −5.8, 95 % CI: −9.2 to −2.4, p = 0.001) and (B = −7.4, 95 % CI: −10.6 to −4.2, p < 0.001).

**Table 4:** Association between the effects of social media on eating behavior score (out of 100 %) and sociodemographic characteristics in the study population (n = 1056).

Parameter	SESMEB score			
		95 % CI		
	B	Lower	Upper	p-value
**Intercept**	32.0	23.9	40.2	**<0.001**
**Age (years)**	−0.3	−0.4	−0.1	**<0.001**
**Sex (reference: female)**				
Male	−5.0	−7.6	−2.5	**<0.001**
**Marital status (reference: married)**				
Single	3.2	0.6	5.8	**0.017**
**Nationality (reference: non-Arab)**				
GCC	−5.8	−9.3	−2.4	**0.001**
Other Arab	−7.4	−10.6	−4.2	**<0.001**
**Employment (reference: unemployed)**				
Employed	0.8	−1.5	3.1	0.495
**Education (reference: university degree or above)**				
High school degree or less than a high school	−0.8	−3.7	2.0	0.563
**BMI (kg/m**^**2**^**)**	0.2	−0.3	0.5	0.086
**Hours on social media (reference: ≥2 h/day)**				
< 2 h/day	−9.5	−11.9	−7.0	**<0.001**
**Following influences (reference: yes)**				
No	−2.5	−4.7	−0.4	**0.022**

CI: Confidence interval; p-values based on a 5 % level of significance following general linear model analyses; Dependent Variable: Score %.

On the other hand, being single was associated with having a 3.2 % higher score (B = 3.2, 95 % CI: 0.6–5.8, p = 0.017). As for employment, BMI, and education level, no significant associations with the effects of social media on eating behavior scores were observed. These findings suggest that age, gender, marital status, nationality, hours spent on social media, and following influences could be crucial factors associated with the effects of social media on eating behavior.

#### 3.4.2 Association between social media's effects on eating behavior and lifestyle habits

Table 5 shows the association between several dietary and lifestyle factors and the SESMEB score using general linear model analyses. The findings indicate that consuming frozen food (B = 12.6, 95 % CI: 3.7–21.5, p = 0.006) and fast food (B = 9.3, 95 % CI: 1.5–17.0, p = 0.020) on most days was associated with higher SESMEB scores which increased by 12.6 % and 9.3 % respectively. Not having breakfast daily was also associated with 3.8 % higher scores (B = 3.8, 95 % CI: 1.3–6.3, p = 0.003). Exercise frequency, household chores, and screen time for work did not show significant associations with the score. However, the amount of screen time for entertainment purposes revealed significant negative associations with the score as less time spent on screens was associated with lower scores. Participants who spent less than 30 min (B = −13.5, 95 % CI: −18.7–8.5, p < 0.001), 1–2 h (B = −8.3, 95 % CI: −11.2 to −5.4, p < 0.001), or 3–5 h (B = −6.0, 95 % CI: −8.6 to −3.3, p < 0.001) on entertainment-related screen time had lower scores by 13.5 %, 8.3 %, and 6.0 % respectively compared to those who spent more than 5 h. These findings suggest that the nature of most consumed meals, the consumption of breakfast, and the time spent on screens for leisure are highly associated with the effects of social media on eating behavior.

**Table 5:** Association between the effects of social media on eating behavior score (out of 100 %) and dietary and lifestyle habits in the study population (n = 1056).

Parameter	SESMEB score			
	B	95 % CI		p-value
		Lower	Upper	
**Intercept**	26.7	13.2	40.2	<0.001
**Consumed meals/week (reference: Healthy restaurants)**				
Homemade	4.8	−2.5	12.1	0.195
Frozen food	12.6	3.7	21.5	**0.006**
Fast food	9.3	1.5	17.0	**0.020**
Restaurants	7.2	−0.9	15.3	0.080
**Consume breakfast (reference: yes)**				
No	3.8	1.3	6.3	**0.003**
**Number of main meals (reference: 5 or more)**				
1 meal	4.7	−6.6	16.1	0.413
2 meals	6.6	−4.1	17.3	0.227
3 meals	7.2	−3.5	17.9	0.188
4 meals	8.9	−2.6	20.4	0.131
**Skipping meals (reference: yes)**				
No	2.3	−0.1	4.6	0.058
**Exercise (reference: every day)**				
Never	−1.5	−5.9	2.8	0.493
1–3 times/week	−1.6	−60	2.8	0.484
4–5 times/week	−1.1	−6.3	4.1	0.668
**Household chores (reference: every day)**				
Never	2.5	−0.3	5.4	0.082
1–3 times/week	1.3	−1.9	4.5	0.419
4–5 times/week	0.5	−4.5	5.4	0.852
**Screen time for work (reference: > 5 h)**				
None	−1.9	−5.0	1.5	0.281
1–2 h	−2.0	−5.0	1.0	0.184
3–5 h	2.3	−0.7	5.4	0.133
**Screen time for entertainment (reference: > 5 h)**				
<30 min	−13.5	−18.7	−8.4	**<0.001**
1–2 h	−8.3	−11.2	−5.4	**<0.001**
3–5 h	−6.0	−8.6	−3.3	**<0.001**

CI: Confidence interval; p-values based on a 5 % level of significance following general linear model analyses. Dependent Variable: Score %.

### 3.5 Changes in lifestyle due to the COVID-19 pandemic

As part of our secondary objective, we set out to investigate whether there was a difference in the SESMEB score according to reported changes in lifestyle and dietary habits due to the COVID-19 pandemic. More than two-thirds of the participants reported being infected by COVID-19 at least once (71.0 %) and 10.1 % of them reported severe illness requiring hospitalization as presented in Table 6. Significant differences in the SESMEB score were observed according to previous infection indicating that those who were ever infected with COVID-19 had significantly higher scores compared to those who were not (p = 0.005).

**Table 6:** The difference in effects of social media on eating behavior score (out of 100 %) according to COVID-19 related changes (n = 1056).

Variable	SESMEB score		
	Mean	SD	p-value^a^
**Previous infection with COVID-19**			0.005
No	34.0	16.6	
Yes	37.3	17.9	
**Hospitalization due to severe infection**			0.361
No	36.9	17.9	
Yes	38.6	17.3	
**Screen time for leisure/entertainment**			<0.001
Decreased	28.4	18.3	
Unchanged	32.6	16.8	
Increased	39.2	17.2	
**Food intake**	30.9 34.0 40.7	16.8 18.0 16.6	<0.001
Decreased			
Unchanged			
Increased			
**Body weight**			<0.001
Decreased	33.1	18.2	
Unchanged	35.5	18.2	
Increased	38.9	16.2	
**Number of meals per day**	32.3 34.0 41.4	17.2 17.8 16.3	<0.001
Decreased			
Unchanged			
Increased			
**Consumption of fried food**			<0.001
Decreased	30.6	16.8	
Unchanged	38.9	18.3	
Increased	41.2	15.6	
**Consumption of fast food**			<0.001
Decreased	30.6	16.3	
Unchanged	36.4	18.3	
Increased	41.3	16.2	
**Consumption of fruits and vegetables**	36.5 35.8 37.2	17.6 18.3 16.2	0.503
Decreased			
Unchanged			
Increased			
**Number of meals with family/friends**			0.003
Decreased	32.9	16.9	
Unchanged	36.9	17.8	
Increased	38.0	17.5	
**Physical activity**			0.137
Decreased	35.5	15.6	
Unchanged	36.4	19.6	
Increased	38.7	17.8	

a p-value was based on the independent T-test and one-way ANOVA at a 5 % level of significance.

**Fig. 5:**
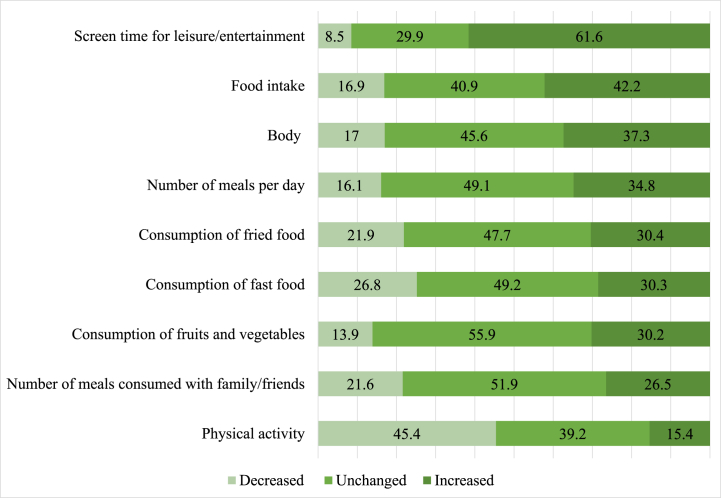
Reported changes in lifestyle due to COVID-19 among study participants (n = 1056).

Fig. 5 presents the effect of the COVID-19 pandemic on certain lifestyle and dietary habits as reported by the participants. The majority of participants reported no change for most of the dietary and lifestyle behaviors; consumption of fruits and vegetables (55.9 %), number of meals consumed with friends and family (51.9 %), consumption of fast food (49.2 %), and number of meals consumed per day (49.1 %). However, most of the participants reported an increase in screen time (61.6 %), and food intake (42.2 %), whereas the majority reported a decrease in physical activity levels (45.5 %). Table 6 shows the differences in SESMEB scores based on reported changes in certain lifestyle and dietary habits due to COVID-19. Overall, the mean score increased significantly as the reported impact of COVID-19 increased in all food items except for the consumption of fruits and vegetables and physical activity. Participants who reported an increase in screen time for entertainment (39.2 ± 17.2), food intake (40.7 ± 16.6), body weight (38.9 ± 16.2), and number of meals per day (41.4 ± 16.3) had significantly higher scores compared to those who reported decreased or unchanged habits. Similarly, those who reported increased consumption of fried food (41.2 ± 15.6), and consumption of fast food (41.3 ± 16.2) had significantly higher scores compared to other categories (p < 0.001). Moreover, those who reported an increased number of meals with family/friends (38.0 ± 17.5) had higher scores compared to those with a decreased or unchanged number of meals (p = 0.003).

## 4 Discussion

The findings of this cross-sectional study among adults in the UAE provide valuable insights related to the possible impact of SMP on eating behavior. Most of the participants reported using social media more than 2 h per day (80.3 %). This was higher than the use reported by undergraduate students in Turkey (54.6 %) [25] and students in Canada (37.9 %) [34]. This is of particular concern since several studies indicate that spending more than 3 h on social media daily increases the risks for mental health issues [35] and suggest that limiting social media use to less than 30 min per day may improve health and well-being [36]. In this study, participants reported mainly using social media to connect, stay informed, for entertainment, looking for places to eat, food recipes, and food reviews. This high level of engagement suggests an important role of social media in the participants' daily lives and a potential impact on their dietary choices.

In the present study, following social media influencers and engaging with promoted diets and food products were prevalent among participants (over 50 %). This is higher than findings in Saudi Arabia where only 36.0 % reported following influencers on social media and around 38.0 % were influenced by promoted foods and diets [32]. This suggests that the phenomenon of following influencers in the UAE is becoming increasingly popular which potentially increases the impact it has on an individual's behavior and attitudes towards diet and food.

In the present study, social media and the internet were more often used as main sources of reference to search for nutrition and nutrition-related problems. This suggests that these platforms are perceived as reliable sources of information and demonstrates the potential for SMP to be used as educational tools to promote healthy eating habits. Previous studies exploring the credibility of health and diet-related information on social media underline the influence of several factors including expertise, message accuracy, and user characteristics. Research shows that people judge the credibility of this information by the author's expertise especially for more health-conscious individuals, while motivation and friendliness are more indicative of credibility for less health-conscious ones [37]. Moreover, a study investigated the credibility of weight management posts by social media influencers, and concluded a lack of credibility in these posts, while the registered nutritionists' posts had the highest credibility score compared to other influencers [38]. In the present study, participants had a high level of trust in nutrition-related information provided by dietitians or nutritionists. This emphasizes the importance of accurate and evidence-based content from accredited sources on SMP to provide consumers with trustworthy information.

In the present study, the SESMEB was used to assess the impact of social media on eating behavior among the participants. Participants mostly reported that social media influenced their views about food, increased their desire to eat, and that refraining from social media was associated with shorter meal duration. This suggests that the visual representation and advertising of different foods on SMP can alter people's attitudes and inclinations toward certain foods. This is supported by evidence from the literature indicating that a longer duration of social media use affects food selection [39] and that higher engagement with food-related content and marketing is associated with a higher intake of unhealthy foods and drinks [40]. This highlights that SMP can trigger cravings and appetites for specific foods. To support these findings, a recent systematic review revealed that exposure to food images and posts on social media was associated with increased body dissatisfaction, increased dieting, overeating, and consumption of some unhealthy foods [24].

Constant exposure to enticing food images and information on social media may boost food cravings and the risk of indulging in certain foods since the majority of food images depicted are energy-dense foods [41]. Furthermore, because many food posts involve specific social situations, such as eating with friends or at restaurants [42], these posts may exacerbate the effect of social media on eating behavior and the desire for food. Therefore, the influence of food-related content on social media emphasizes the relevance of addressing the purpose of these platforms in affecting eating behavior and food preferences.

The mean SESMEB score in this study was 44.15 ± 12.68 (range 18–90), corresponding to 36.3 % upon data transformation. The results of the general linear model analyses revealed multiple significant relationships between sociodemographic and social media use characteristics and the SESMEB score. The score was significantly lower among males compared to females, suggesting a higher influence of social media on females’ eating behavior. This is consistent with a previous study conducted in Saudi Arabia, which found that males were less likely to be affected by social media in terms of their eating behaviors [32]. Available evidence indicates a strong negative correlation between age and social media use [43]. In the present study, a significantly lower SESMEB score was associated with older age and being married, suggesting that the eating behavior of younger individuals and those who are single may be potentially more influenced by social media. Similar findings were reported among adults in Saudi Arabia, where younger and single participants were almost twice as likely to have a higher SESMEB score compared to their counterparts [32]. Moreover, in the present study, participants from the GCC and other Arab countries scored significantly lower than non-Arabs, suggesting that cultural and regional factors may combine with social media use and influence eating behavior differently in various groups.

Spending more time scrolling through social media and following influencers was significantly associated with a higher effect on eating behavior among our participants. These findings highlight how the duration of SMP use and the exposure to the content shared by influencers may have a strong impact on the extent of the influence of social media on eating behavior. These findings are in line with other studies showing that being a female and spending extended time using social media is associated with a higher influence on eating behavior as well as emotional eating [25]. Additionally, in another study, following influencers was associated with a 10 times higher likelihood of that influence [32]. These findings contribute to the current knowledge about the complicated interplay between sociodemographic characteristics, social media use, and their impact on people's eating behavior.

The findings of this study reveal several interesting links between lifestyle and dietary factors and the effect of social media on eating behavior. Consuming foods other than homemade ones on most days, and not consuming breakfast was associated with significantly lower SESMEB scores. This suggests a potentially harmful influence of social media on dietary habits, where such habits are often linked with unhealthy eating behavior. The current findings are in line with a study among Canadian students revealing the use of SMP was associated with higher odds of skipping breakfast and consuming sugar-sweetened beverages [44]. Moreover, the present study analysis revealed that as time spent using screens for entertainment increased, the effect of social media on eating behavior increased. This is expected as available evidence indicates that sedentary behavior, and in particular high screen time is an important predictor of unhealthy eating habits [45], and that screen use while eating may increase food intake [46]. These findings emphasize the necessity of taking dietary and lifestyle factors into account when studying the impact of social media on eating behavior. Future studies should look at the underlying mechanisms that interact with social media use, as well as potential interventions to improve healthier eating habits in the online environment.

Research amid the COVID-19 pandemic revealed several changes in people's dietary and lifestyle habits including, but not limited to, increased body weight, sedentary behavior, screen time, and unhealthy dietary habits [47]. This persisted even after the availability of the COVID-19 vaccination and after the pandemic pressures subsidized [29]. Building upon these previous findings, a secondary objective in the research was to extend our understanding by investigating a link between social media use and eating behaviors. In this study, participants who were ever infected with COVID-19 had significantly higher SESMEB scores compared to those who were not, suggesting that previous experience with a COVID-19 infection may be associated with a higher influence of social media on eating behavior. Moreover, an upward trend was observed when comparing the SESMEB score and reported changes in lifestyle and dietary habits. As participants reported an increased food intake, body weight, number of meals, consumption of fast or fried food, and screen time, the score increased significantly compared to those reporting decreased or unchanged behaviors. Previous research suggested a protective role of family-shared meals during COVID-19 on dietary intake and emotional well-being [48]. Interestingly, the present study reported an increase in shared meals with family, which coincided with significantly higher SESMEB scores. This points to a possible relationship between changes in meal-sharing dynamics due to the pandemic and the impact of social media on eating behavior. Nonetheless, while the findings indicate significant differences in SESMEB scores and changes due to the COVID-19 pandemic, it is important to interpret these findings with caution. They provide insights into how perceived changes in lifestyle due to the pandemic may be related to the effect of social media on eating behavior and lay the groundwork for further investigation into potential long-term links between COVID-19, social media, and eating behavior.

## 5 Study limitations

To the best of our knowledge, this is the first study to assess the association between social media and eating behavior in the UAE. While the cross-sectional design helps provide valuable insights into factors that may be associated with the effect of SMP among the study population, some limitations are implied. The nature of the study design limits the ability to establish causal relationships. Moreover, the dependence on self-reported information is subject to recall bias and social desirability bias. In addition, the web-based recruitment and survey link may introduce selection bias, impacting the generalizability of the findings. Our research focused on adults aged 18 and above actively using social media in the UAE, which inherently limits generalization. Inclusion criteria did not encompass factors like specific dietary needs, mental health status, or ethnicity, which could influence social media usage patterns. Given the UAE's diverse ethnic composition from high immigration rates, our findings may not fully represent all demographic experiences. Future studies could benefit from broader inclusion criteria to comprehensively analyze social media behaviors in the UAE. Moreover, our study did not include adjustments for confounding variables in the statistical analyses, which may have introduced biases in the observed associations between social media use and eating behaviors. Further, given the retrospective nature of the questions regarding lifestyle and dietary changes during the COVID-19 pandemic, there is a potential for recall bias. Participants may not accurately remember their specific behaviors and dietary habits from two or more years ago. As our questions focused on broad trends rather than precise quantities, respondents may rely on general impressions or significant events rather than specific details. This limitation could affect the accuracy of the data related to changes in eating behaviors and lifestyle habits. Consequently, the findings related to the impact of the pandemic on these behaviors should be interpreted with caution. Nonetheless, the study included a large sample size with reasonable distribution among the UAE emirates which helps enhance the generalizability of the results to the target population. Also, recruiting participants using several approaches helped minimize the self-selection bias. Lastly, the use of a novel validated, and reliable scale further enhanced the credibility of the findings. Overall, the study provided important and needed insights into the potential impact of SMP on people's eating behavior, especially at a time when social media has become an integral part of people's lives.

## 6 Conclusion

This study highlights the extent of the effect of SMP on people's eating behavior and provides important insights into its association with lifestyle habits and the use of social media as well as the potential effect of the COVID-19 pandemic on people's lifestyle and dietary habits. The findings revealed that being exposed to food on SMP may affect how people perceive food and how it could act as a stimulant to consume foods. However, the effect on actual food consumption is yet to be explored. Certain sociodemographic characteristics and trends of social media use, such as younger age, being a female, and increased time spent on social media appeared to increase that effect. The COVID-19 pandemic has also been shown to affect participants' dietary and lifestyle habits, and a higher score of the social media effect on eating behavior was recorded for those reporting increased food intake, body weight, and the number of meals due to the pandemic.

## Future research

There is no doubt that technological advancements nowadays are penetrating multiple aspects of people's lives and will continue to do so in the upcoming years. The study aims to contribute both theoretically and practically to understanding the impact of social media on eating behaviors among adults in the UAE. The theoretical contribution lies in expanding knowledge about how social media influences dietary habits, particularly in a region with limited prior research on this topic. Our study offers insights into the associations between social media use, sociodemographic characteristics, and lifestyle habits. Practically, our findings are intended to inform public health practitioners, policymakers, and educators about the potential effects of social media on dietary behaviors. Specifically, understanding which demographic groups and lifestyle patterns are more susceptible to these influences can guide targeted interventions to promote healthy eating habits and mitigate negative effects associated with excessive social media use. Future research directions should include establishing targeted programs aimed at increasing awareness, mitigating the effect, and advocating for a positive impact of social media on people's daily lives to encourage people to eat healthily. Moreover, future research could investigate the impact of SMP on actual food consumption behavior and consider psychological and social factors for a more comprehensive viewpoint. Furthermore, governments, health organizations and healthcare professionals must advocate for promoting healthy dietary habits among social media users. Collaborations with social media influencers in providing more reliable and evidence-based nutritional and health content on these platforms could be also a promising strategy.

## Ethics approval and consent to participate

This study was reviewed and approved by the Research Ethics Committee at the University of Sharjah with the approval number: REC-23-03-22-01-S, dated March 22, 2023. All participants/provided written informed consent to participate in the study and for their data to be published.

## Funding

This research received no specific grant from any funding agency, commercial or not-for-profit sectors.

## Data availability statement

Data associated with the study has not been deposited into a publicly available repository. Data are available from the corresponding author upon reasonable request.

## CRediT authorship contribution statement

**Leila Cheikh Ismail:** Writing – review & editing, Writing – original draft, Validation, Methodology, Formal analysis, Conceptualization. **Tareq M. Osaili:** Writing – review & editing, Writing – original draft, Validation, Methodology, Formal analysis, Conceptualization. **Farah Naja:** Writing – review & editing, Validation, Methodology, Conceptualization. **Mary Wartanian:** Investigation. **Gadeer Elkabat:** Investigation. **Mariam Arnous:** Investigation. **Hala Alkoukou:** Investigation. **Maysm N. Mohamad:** Writing – review & editing, Validation, Methodology. **Sheima T. Saleh:** Writing – original draft, Formal analysis. **Rameez Al Daour:** Investigation. **Emad Masuadi:** Writing – original draft, Formal analysis. **Habiba I. Ali:** Writing – review & editing. **Lily Stojanovska:** Writing – review & editing. **Ayesha S. Al Dhaheri:** Writing – review & editing.

## Declaration of competing interest

The authors declare that they have no known competing financial interests or personal relationships that could have appeared to influence the work reported in this paper.
