# Supplementary material for: The association of social media with dietary behaviors among adults in the United Arab Emirates
Source: Heliyon. 2024 Jul 31;10(15):e35574. doi: 10.1016/j.heliyon.2024.e35574 (PMC11334855; doi:10.1016/j.heliyon.2024.e35574)
Supplement: Multimedia component 1 [file mmc1.docx]

**The effect of social media on dietary habits due to the COVID-19 pandemic**

# **Socio-demographic information**

1. **What is your gender?**
2. Female
3. Male
4. **How old are you in years?**

- ____________

1. **Which emirate do you reside in?**
2. Abu Dhabi
3. Dubai
4. Sharjah
5. Ajman
6. Umm Al Quwain
7. Ras Al Khaimah
8. Fujairah
9. **What is your nationality?**

- Emirati citizen
- Arab (GCC: Bahrain, Kuwait, Oman, Qatar, Saudi Arabia)
- Arab (other countries)
- Non-Arab

1. **What is your marital status?**

- Single
- Married
- Divorced/Widowed

1. **What is your education Level?**

| - High school or less |
| --- |
| - College/Diploma |
| - Bachelor’s degree |
| - Higher education MSc. /Ph.D. |

1. **What is your employment status?**

- Employed
- Student
- Unemployed

1. **What is your current weight? __________(kg)**
2. **What is your height? ___________ (cm)**

# **Use of social media**

1. **How much time do you spend on social media (hours/day)?**

- ≤2 h/d
- >2 h/d

1. **Why do you mainly use social media?** *(you can choose more than one option)*

- Connecting with friends and family
- Staying informed (following news and updates)
- Sharing content (share thoughts, opinions, and experiences with others)
- Working (building a brand, connecting with clients or employers, marketing)
- Entertainment (watching videos, playing games)
- For studying/research purposes
- Other, _______________

1. **What type of social media do you usually use?** *(you can choose more than one option)*

- Instagram
- Twitter
- Tiktok
- Youtube
- Facebook
- Snapchat
- Pinterest
- LinkedIn
- Other _________________

1. **When looking for food references, what type of social media do you usually use?** *(you can choose more than one option)*

- Instagram
- Twitter
- Tiktok
- Youtube
- Facebook
- Snapchat
- Pinterest
- LinkedIn
- Other _________________

1. **What type of information on social media do you look for related to food?** *(you can choose more than one option)*

- Food reviews
- Places to eat
- Food recipes
- Food promotion
- Food and nutrition content

1. **Are you interested in nutrition news on social media?**

- Yes
- No
- Partially
  1. **If yes or partially, why do you follow nutrition news on social media?** *(you can choose more than one option)*
- Learning to eat healthier food
- To follow current information
- Learning to eat in diseases
- Weight control and weight loss
- Learning healthy recipes

1. **What is the main source you refer to in any nutritional problem?**

- Internet/social media
- Dietitian
- Doctor
- Food engineer/specialist
- Another __________________

1. **Do you pay attention to the sources of nutrition-related posts on social media?**

- Yes
- No
- Sometimes

1. **Which of the following do you think is a factor that influences the reliability of nutrition-related posts on social media?**

- Written by a nutritionist/dietician
- Written by the doctor or healthcare professional
- Written by someone who shares their experiences (losing weight, fighting disease, etc.)
- Others

1. **Do you follow influencers on social media?**

- Yes
- No ***(skip to the next section)***
  1. **If yes, have you ever tried a diet promoted by a social media influencer?**
- Yes
- No
  1. **f yes, have you ever bought a food product that was promoted by a social media influencer/ celebrity?**
- Yes
- No

# **Effects of social media on eating behavior**

| **Statement** | **Never** | **Rarely** | **Sometimes** | **Often** | **Always** |
| --- | --- | --- | --- | --- | --- |
| 1. The inclusion of food on social media influences my view of that food |  |  |  |  |  |
| 1. I see and consume any food on social media that are not my food habit |  |  |  |  |  |
| 1. Even though I’m full, I eat food or dishes I see on social media |  |  |  |  |  |
| 1. I think that the foods on social media are more beneficial for health |  |  |  |  |  |
| 1. After I started using social media, my fast-food/cook-chill food consumption increased |  |  |  |  |  |
| 1. I follow nutrition news/blogs/pages on social media |  |  |  |  |  |
| 1. Without getting tired I buy/cook a food/dish that I see on social media |  |  |  |  |  |
| 1. I regulate my diet according to shared news/photos/videos about the foods/dishes I see on social media |  |  |  |  |  |
| 1. I am constantly snacking when surfing on social media, and I realize how much I’ve eaten later |  |  |  |  |  |
| 1. I am interested in foods/dishes shared by celebrities on social media and I consume that food/dish |  |  |  |  |  |
| 1. If I did not use social media, my time for eating would be reduced |  |  |  |  |  |
| 1. When surfing on social media, even though I am full I am snacking |  |  |  |  |  |
| 1. I consume foods/dishes shared by people who have a lot of followers on social media |  |  |  |  |  |
| 1. I think foods or dishes with more likes or shares on social media are healthier |  |  |  |  |  |
| 1. The foods/dishes that I see on social media arouse my desire to eat |  |  |  |  |  |
| 1. I consume foods or dishes that have more news or photo and video likes on social media |  |  |  |  |  |
| 1. I think that foods or dishes with more likes or shares on social media are more reliable |  |  |  |  |  |
| 1. On the days I use social media for a long time, my desire to eat increases and I eat more |  |  |  |  |  |

# **Dietary habits**

1. **Most of your consumed meals during the week are?**

- Homemade
- Frozen ready-to-eat meals
- Fast food
- Restaurants
- Healthy food Restaurants

1. **Do you consume breakfast daily?**

- Yes
- No

1. **How many main meals do you eat per day?**

- 1
- 2
- 3
- 4
- 5 or more

1. **Do you usually skip meals?**

- Yes
- No
  1. **If yes, why is that?**
- To reduce food intake
- Lack of time
- To lose weight
- Lack of appetite
- Fasting

1. **How many snacks (between meals) do you have per day?**

- 0
- 1
- 2
- 3
- 4 or more

1. **How much water do you drink daily?**

- Less than 8 cups (< 2 liters)
- 8 cups or more (≥ 2 liters)

1. **How often do you eat the foods listed below?**

| **Food Item** | **Never** | **1-4 times/week** | **Once/day** | **2-3 times/day** | **4 or more times/day** |
| --- | --- | --- | --- | --- | --- |
| Fruits |  |  |  |  |  |
| Vegetables |  |  |  |  |  |
| Milk and milk products |  |  |  |  |  |
| Meat/Chicken/Fish |  |  |  |  |  |
| Bread/rice/pasta |  |  |  |  |  |
| Sweets/ desserts |  |  |  |  |  |
| Coffee/Tea |  |  |  |  |  |
| Sweet drinks (soft drinks, canned juice, etc.) |  |  |  |  |  |
| Energy drinks |  |  |  |  |  |

# **Physical activity**

1. **Do you do any exercise (walking, running, equipment, etc.)?**

- Never ***(skip to Question 2)***
- 1-3 times/week
- 4-5 times/week
- Everyday

1. **Do you do household chores?**

- Never
- 1-3 times/week
- 4-5 times/week
- Everyday

1. **How much time do you spend on the computer for work/ study daily?**

- None
- 1-2 hours
- 3-5 hours
- More than 5 hours

1. **How much time do you spend daily on Television, computer, social media for entertainment?**

- Less than 30 minutes
- 1-2 hours
- 3-5 hours
- More than 5 hours

# **COCID-19 related questions**

1. **Have you been infected by the COVID-19 disease?**

- Yes
- No
  1. **If yes, were you hospitalized?**
- Yes
- No

1. **Please answer the following questions how the COVID-19 affected your daily habits**

|  | **Decreased** | **Unchanged** | **Increased** |
| --- | --- | --- | --- |
| - 1. Your body weight |  |  |  |
| - 1. Your food intake was |  |  |  |
| - 1. Number of meals you eat per day |  |  |  |
| - 1. Your consumption of fruits and vegetables |  |  |  |
| - 1. Your consumption of fast foods |  |  |  |
| - 1. Your consumption of fried foods |  |  |  |
| - 1. The number of meals you consume with family or friends |  |  |  |
| - 1. Your physical activity level |  |  |  |
| - 1. Your screen time for leisure /entertainment |  |  |  |
